# Tragic choices in intensive care during the COVID-19 pandemic: on fairness, consistency and community

**DOI:** 10.1136/medethics-2020-106487

**Published:** 2020-08-07

**Authors:** Chris Newdick, Mark Sheehan, Michael Dunn

**Affiliations:** 1 School of Law, University of Reading, Reading, Berkshire, UK; 2 Ethox Centre, University of Oxford, Oxford, UK; 3 Wellcome Centre for Ethics and the Humanities, University of Oxford, Oxford, UK; 4 Oxford NIHR Biomedical Research Centre, Oxford University Hospitals Trust, Oxford, UK

**Keywords:** allocation of health care resources, decision-making, clinical ethics, law

## Abstract

Tragic choices arise during the COVID-19 pandemic when the limited resources made available in acute medical settings cannot be accessed by all patients who need them. In these circumstances, healthcare rationing is unavoidable. It is important in any healthcare rationing process that the interests of the community are recognised, and that decision-making upholds these interests through a fair and consistent process of decision-making. Responding to recent calls (1) to safeguard individuals’ legal rights in decision-making in intensive care, and (2) for new authoritative national guidance for decision-making, this paper seeks to clarify what consistency and fairness demand in healthcare rationing during the COVID-19 pandemic, from both a legal and ethical standpoint. The paper begins with a brief review of UK law concerning healthcare resource allocation, considering how community interests and individual rights have been marshalled in judicial deliberation about the use of limited health resources within the National Health Service (NHS). It is then argued that an important distinction needs to be drawn between procedural and outcome consistency, and that a procedurally consistent decision-making process ought to be favoured. Congruent with the position that UK courts have adopted for resource allocation decision-making in the NHS more generally, specific requirements for a procedural framework and substantive triage criteria to be applied within that framework during the COVID-19 pandemic are considered in detail.

What happens if demand for intensive care exceeds the treatment facilities available? How should doctors decide between us? The COVID-19 pandemic has made these questions pressing, and across the world much attention has been paid to the actual decisions that doctors are expected to make. As of May 2020, professional bodies have invested much effort in generating guidance of various forms on how these decisions should be made—in almost all cases the guidance seems to permit unpalatable decisions about who gets treatment.^1 2^ As we write, the UK is coping with the additional demands placed on it without extensive rationing of ventilators or intensive care beds. However, the experience clearly highlights the need for more systematic responses associated with the need to prioritise and deprioritise a wide range of interventions during the pandemic.

COVID-19 has provoked two main responses from ethicists and lawyers concerned with medical decision-making. For some, emerging and broad ethical guidance produced by professional bodies does not go far enough. What is needed, so the claim goes, is overarching, national guidance from government that commands authority and ensures consistent, fair decision-making.^3 4^ For others, the response to the guidance produced has been the assertion of individual rights against rationing, warning that any compromise of normal treatment is vulnerable to legal challenge, and should be undertaken with caution for fear of overlooking patients’ rights.^5^

We take issue with both these views. Rationing decisions should certainly not be ad hoc, but an individual’s ‘right to treatment’ has meaning only in the context of the community in which rights are valued. Indeed, an overly ‘individualistic’ approach to rights damages health. Healthcare rights may be imprecise and come into conflict, but they never contradict community interests. This is because rights always belong within the scope of things that are of value for the community that recognises them. In this sense, because the protection of individual rights matters for the community, it is in the interests of the community to safeguard them in a way that is consistent with other things that are of value.^6 7^ This pandemic provides an opportunity to rebalance the debate about healthcare rationing generally, as well as how notions of authority and fairness in decision-making concerning the use of resources in the National Health Service (NHS) ought to be configured.^8^

Our starting point, therefore, is ‘community’, properly understood. We reject the notion that individual rights have some special status independent of the community in which they are recognised.^9–11^ We argue that in law and ethics the fundamental objective of any decision to ration health resources is *to serve the best interests of the community as a whole by responding to patients fairly, equally and consistently*. In order to do this, group interests need to be balanced against the interests of specific individuals. Rights are not overlooked by balancing the needs of those already in intensive care with those awaiting admission because of capacity constraints, but it is crucial to be clear about how this process of balancing is achieved.

We progress as follows: in (A) Balancing Individual and Public Interests section, we outline a series of legal principles on the balance between individual and community interests, in the context of judicial review, human rights and healthcare rationing in the UK. In (B) Procedural versus Outcome Consistency section, we distinguish between procedural and outcome consistency. In (C) Procedural Framework section, we describe the procedural framework necessary to promote fairness and consistency, and in (D) Substantive Triage Criteria section, we consider a range of substantive triage criteria that give practical effect to the balancing of individual and community interests.

## Balancing individual and public interests

In considering how interests in healthcare can be conceived at the time of a pandemic in the UK, it is important to bear in mind the following four legal principles:

(1) *Claims to treatment from single claimants must always be balanced against ‘the rights of others’.*

Healthcare policy may benefit some and harm others. But even here, in a series of cases, the European Court of Human Rights has refused to guarantee treatment to those adversely affected. For example, in *Pentiacova v Moldova*,^12^ patients suffering from chronic renal failure complained that their haemodialysis services were being rationed, to the detriment of their health. The European Court of Human Rights rejected their claim. It said:

The applicants’ claim amounts to a call on public funds which, in view of the scarce resources, would have to be diverted from other worthy needs funded by the taxpayer… While it is clearly desirable that everyone should have access to a full range of medical treatment, including life-saving medical procedures and drugs, the lack of resources means that there are, unfortunately, in the Contracting States many individuals who do not enjoy them, especially in cases of permanent and expensive treatment… [I]t cannot be said that the respondent State failed to strike a fair balance between the competing interests of the applicants and the community as a whole.

For example, resource allocators should consider the relative need for treatment, the likely benefit, equality of access, evidence of efficacy and cost. In respect of COVID-19 treatment, relevant too may be the disproportionate impact on black, Asian and minority ethnic communities. As the science develops, so the response should be reasonable and proportionate. Decisions of this gravity cannot be left to individual doctors alone; a framework of procedural guidance is required. This might be created by clinicians, hospital managers and patient representatives to optimise available resources, perhaps administered by triage coordinators and (ideally) an appeal mechanism.

(2) *Judicial review of resource allocation acknowledges that hard choices are inevitable.*

As the Court of Appeal said in *R v Cambridge DHA*,^13^ ‘Difficult and agonizing judgments have to be made as to how a limited budget is best allocated to the maximum advantage of the maximum number of patients. That is not a judgment the court can make.’ The court has neither the authority nor expertise to do so, even though judicial review subjects their rationality to close scrutiny. It demands that public authorities consider all the relevant factors, including the resources available. This explains why a procedural framework is so crucial to this process (see ref 14). But it does not undermine the central principle that rationing is lawful—provided it responds to everyone’s need fairly and consistently. This creates *procedural* rights to a fair system of decision-making, but not *substantive* rights of access to particular treatment.

COVID-19 has not (yet) required doctors to ration intensive care. If limited resources were to cause conflict, patients in intensive care could not claim greater rights to treatment than urgent patients awaiting admission. In such a case, treatment could not simply be withdrawn. However, as the National Institute for Health and Care Excellence (NICE) says, doctors could lawfully prioritise care (say) by moving a patient from an intensive ward to a high-dependency, or step-down unit, or transfer the patient to intensive care elsewhere.^15^ That said, decisions which involve life and death, or that damage health, will be subject to careful scrutiny to ensure relevant factors have been weighed and balanced properly.

(3) *Precisely this approach has been confirmed in a case involving COVID-19.*

On 9 April 2020, in *University College London Hospitals NHS Foundation Trust v MB*,^16^ the High Court considered a decision of a Foundation Trust to *withdraw* residential care from a mental health patient so as to make space to accommodate other patients with COVID-19. The court upheld the decision. It said, at para 55:

In some circumstances, a hospital may have to decide which of two patients, A or B, has a better claim to a bed, or a better claim to a bed in a particular unit, even ceasing to provide in-patient care to one of them… will certainly cause extreme distress or will give rise to significant risks to that patient’s health or even life. A hospital which in those circumstances determines rationally, and in accordance with a lawful policy, that A’s clinical need is greater than B’s, or that A would derive greater clinical benefit from the bed than B, is not precluded by Article 3 ECHR from declining to offer inpatient care to B. This is because in-patient care is a scarce resource and, as Auld LJ put it in R v North West Lancashire Health Authority ex p. A [2000] 1 WLR 977, at 996, ‘[i]t is plain… that article 3 was not designed for circumstances… where the challenge is as to a health authority’s allocation of finite funds between competing demands.’ Decisions taken by a health authority on the basis of finite funds are, in my judgment, no different in principle from those taken by a hospital on the basis of finite resources of other kinds. In each case a choice has to be made and, in making it, it is necessary to consider the needs of more than one person.

As the Court of Appeal has confirmed, our right of patient choice arises only within the range of treatments that are reasonably available to us although, as we note above, if such a decision to benefit one patient were to expose another patient to significant risk, the decision could be subject to close scrutiny in judicial review.^17^

(4) *The statutory duty to allocate resources belongs to the relevant public authority, not judges. Public authorities have wide discretion in making resource allocation decisions.*

As the Supreme Court has said, the European Convention on Human Rights confers broad discretion on public authorities allocating resources ‘between the competing interests of the individual and of the community as a whole’.^18^ The court’s powers are no greater than those available to applicants in judicial review. In *N v ACCG*,^19^ the applicant family sought the resources necessary to be trained as carers providing round-the-clock care for their disabled son at home. It might have been in his best interests (or not), but the clinical commissioning group (CCG) could not afford to adopt such a policy for everyone and refused the request. The parents’ application was refused. Such a decision involves issues of affordability and the interests of other patients unknown to the court. For this reason, this was not a decision for judges. Resource allocation is for the CCG. At paras 35–44, the Supreme Court said:

So how is the court’s duty to decide what is in the best interests of [the patient] to be reconciled with the fact that the court only has power to take a decision that [the patient] himself could have taken? It has no greater power to oblige others to do what is best than [the patient] would have himself [ie, in JR]. This must mean that…the court can only choose between the available options… the court did not have power to order the CCG to fund what the parents wanted.

These principles bear on the crisis produced by COVID-19 in the UK. In this national context, individual rights can be understood only within the constraints on public resources. In extreme cases, resources may be withdrawn from one patient to divert treatment to others. This decision rests with the public authority, not the court. The attraction of consistency should not encourage triage criteria to undervalue (say) elderly patients as a group, or patients with disabilities. Our individual needs and circumstances must be balanced with those of others without prejudgement by ‘group-stereotyping’. Nevertheless, provided the process balances the relevant circumstances fairly and proportionately and with proper regard for equality and human rights, the decision will be upheld.^20^

The challenge, therefore, is to understand how to guarantee that these difficult and unenviable decisions respond fairly, equally and consistently to patients. This is the purpose of the procedural framework which we outline below.

## Procedural versus outcome consistency

How should doctors decide between us? Inevitably, intensive care doctors may differ—even about the same patient. Some may insist on their Hippocratic duty to promote the best interests of their individual patients. Others may promote the greatest benefit for the greatest number and create space to help those most likely to recover quickly. Yet others may favour the first in the queue, or some over others (eg, clinicians, care workers or delivery drivers), or disfavour patients over a certain age. Still others may adopt a provisional Hippocratic approach unless and until they are given clear instructions to the contrary by their hospital employer. Importantly each of these variations represents distinctive but nevertheless reasonable interpretations of what it means to be a clinician and perform the professional role of the doctor. These different views may represent what individual clinicians take to lie at the very heart of what it is to be a doctor.

Such variation, even if it is reasonable and the product of genuine and deep commitment, is unsettling: it clearly leads to inconsistent responses to the rationing context presented by COVID-19 which look unfair and unattractive. As has been claimed, decision-making about life and death surely requires consistency.^3 4^ The value of consistency in decision-making is not just important for patients; doctors too will want reassurance that they are acting ethically and legally, otherwise an absence of proper guidance exposes clinicians to complaint afterwards. However, those calling for national guidance in order to provide such consistency need to address an important question: what does consistency demand of doctors and others in this context?

In our view, national guidance would encourage consistent decision-making. But, as desirable as national guidance would be, it is not strictly necessary. It is true that the government may have an obligation under the European Convention of Human Rights to establish a national framework of guidance if a public authority exposes people to systematic danger which cannot be managed locally. Until that time, however, we note that priority setting decisions within CCGs have never had the benefit of overarching national guidance because the government has taken the view that local decision-makers are best able to respond to local needs. Instead, judicial review has clarified how local decision-makers should introduce fair and consistent procedures to be legally defensible. What is essential is for a framework to spell out which values are to be considered in funding decisions and how they are to be considered in ways that are fair, rational and consistent.^21–25^

It is helpful, in this context, to distinguish procedural and outcome consistency.^26^ Understandably, both patients and clinicians find appeal in the idea of outcome consistency: guidance that operated in this way would enable people to know where they stand, in advance of the decision being made. An algorithmic or formula-based decision-making process that sought to crunch the relevant values and facts would likely lie at the heart of guidance of this kind. One reason that people find this appealing might lie in the fact that providing a kind of metric or formula makes hugely difficult problems seem more manageable. But of course, such an approach disguises the difficulty and hides it behind the algorithm so that we may be encouraged to expect outcome consistency in circumstances where it is impossible to achieve.

In contrast, national guidance that embraced procedural consistency would require us to accept that these difficult decisions depend irreducibly on the details of the case and the context of the decision. This is not to accept that anything goes, however. We need assurance that the relevant facts and values have been given reasonable consideration in a more open-ended process of decision-making. Crucially, procedural consistency means that the set of things considered, and the way they have been considered, is the same (or very similar) across cases without prejudging the individual elements of the case. Procedural consistency means accepting that a consistent process can give rise to different and so seemingly inconsistent outcomes: we might reasonably differ about whether a person should be allocated an intensive care bed or a ventilator when we have balanced all the relevant factors in this judgement. Inevitably, therefore, although the procedures between different decision-makers may be analogous, the *outcomes* between them may legitimately differ, according to local and individual need, and the availability of resources.

## Procedural framework

In our view, healthcare resource allocation should promote procedural consistency, rather than outcome consistency. This is not to say that outcome consistency is irrelevant. Instead, it is that the latter is unachievable in decisions of this kind for the reasons that we discussed above. A procedural framework should consider how decisions governing resources allocation are made, sensitive to the legal requirements discussed above in 'Balancing Individual and Public Interests'. Importantly, such a framework sits above, and functions to structure, local context-specific decisions ensuring that the interests of individual patients as well as the community are accorded proper respect. A framework of reasonable procedures is an essential means of expressing the community’s interest in responding to everyone’s needs fairly and consistently.

There are two main reasons for adopting this approach that apply equally to triaging decisions during a pandemic, as well as to other prioritisation decisions that need to be made. First, the ethical values that underpin fairness in decision-making are reasonably contested; there is no single ethical position about the nature and extent of resources people are entitled to receive. We can reasonably disagree whether to maximise utility or equity (or clinical freedom). We can reasonably disagree about whether health professionals should be prioritised, or those who have lived with social and economic disadvantage. Second, the resources context within which the decision is made is morally significant. Precisely how we interpret these considerations, or accord them weight, will depend on the circumstances of each healthcare setting and each individual patient.

How, then, should procedural consistency be realised in any local process of triage decision-making endorsed within national guidance? As others have argued,^27 28^ and consistent with the legal and ethical requirements of procedural consistency in resource allocation decision-making generally, we encourage hospitals to appoint a team of senior doctors, perhaps with an independent member, a ‘triage team’, to oversee and support clinical decision-making and, if needs be, make final decisions about individual patients. This will promote procedural consistency in the decision-making process. Further, as hospitals seek to coordinate their activities with one another at regional level, this internal hospital system should be reviewed regionally, to ensure that the requirements of consistency are met. Admittedly, this process diverts senior doctors from treating patients. It will be time consuming and emotionally draining. There is a difficult balance between providing reasonable procedural overview and letting doctors do their job. However, when searching legal and ethical questions are asked in future, the risk of inconsistency from not having such a system is obvious for both patients and doctors.

In addition to notions of fairness and consistency, an important part of this procedural framework concerns the duty of transparency and candour to patients. As is now well established, the principles of informed consent require clinicians to explain to patients and relatives both the uncertainties about their care and the processes in place for making decisions, including in extreme circumstances, the difficult decision to withdraw or withhold treatment.^29–31^

## Substantive triage criteria

As we have seen, procedural consistency does not abrogate responsibility to determine the substantive criteria for decision-making about patients within the agreed framework. Understandably, a consistent decision-making process requires content—those ethical values which shape and inform all reasonable decisions. What substantive triage criteria should be used to distinguish between patients and how should they be incorporated into a procedural framework for fair decision-making?

Clearly, this is where much of the debate has taken place. On our account, it is also the place where all relevant concerns are heard and balanced together. Once we give up on the quest for outcome consistency, the task is to ensure that all the individual features that matter morally are included, and that those that are discriminatory, irrelevant or non-applicable in context are not. Put another way, the guidance should capture all and only the ethically relevant features that go into the decision. Striking a reasonable balance here between specifying broad values or considerations, and determining specific criteria, is a challenge.

How generic or patient specific then should the criteria be? The Belgian Society of Intensive Care recommended that elderly residents in retirement homes should not normally be admitted to intensive care because it would be ‘disproportionate’.^32^ This seems a very broad criterion. Many dependent residents retain significant independence and require limited care. It might be criticised for failing to consider the circumstances of individuals. An analogous concern was raised in March 2020, when NICE recommended use of the Clinical Frailty Score (of 1–9). The recommendation was criticised by MENCAP and other non-governmental bodies for being too generic. Shortly afterwards, NICE acknowledged it had failed to indicate that not all patients on the same point of the frailty scale were the same. For example, those with stable mental illness should not be considered the same as dependent adults whose conditions were deteriorating.^33^

So, should clinical triage criteria be more patient specific? The Pittsburgh Sequential Organ Failure Assessment scoring system attempts to achieve this by putting numbers against particular conditions (on a scale of 1–8, with the most severely ill patients scoring the highest number).^34^ The express aim is to exclude some patients in order to admit others and promote the survival of the greatest number. Those with comorbidity likely to end their lives within 1 year are scored more highly than those likely to live for a further 10 years. This scoring system is helpful as a general guide to distinguish classes of patients, but how far can it take us towards distinguishing at the level of individual patients? Bear in mind a number of variables, for example, patients often present with a mixture of comorbidities, some more severe, others less. Say we suffer a combination of diabetes, hypertension, obesity and heart disease. Comparing one patient to another on a standard scale is unlikely to capture the subtle differences of clinical condition between us. Second, different doctors may assess the prognosis and diagnosis of the same patient differently. Third, the availability of intensive care beds may vary from time to time so that even patients with identical scores may not receive identical care at different points in time. Finally, getting clear guidance from patients themselves about their own wishes may be extremely difficult. Thus, the unique nature of individual circumstances limits the reach of scoring systems in any defensible procedural framework. Indeed, it is misleading to suggest they provide an ‘objective’ decision-making tool or algorithm at the level of individual patients. Uncertainty and disagreement between doctors are inevitable and reasonable discretion within the familiar *Bolam* 35 test is always likely to play a role.^1^

Where is the balance between systems which are too generic and those so precise as to camouflage reality? There are compromises to be made all along the spectrum. An alternative approach is not to use numbers but, instead, to place patients into broad categories, perhaps based around high, medium and low priority.^2^ At highest priority would be those with the highest probability of making a rapid recovery. Low priority is given to patients with the lowest probability of doing so. Priority is given to patients according to their capacity to recover and the speed with which they are likely to do so. On this view, patients are not assigned a ‘score’—this is not an algorithm. Instead, doctors are responsible for applying flexible criteria within their own reasonable clinical discretion, together with their triage teams and coordinators, in the light of each patient’s individual circumstances and the resources available. The advantage of such a system is that it does not promise more ‘objectivity’ in the selection criteria than can be delivered; it does not promise, in advance of the actual context, more determination than is possible or ethically justifiable. It goes without saying that proper records of every such decision must be maintained so that we can understand how and why each decision was made.^36^ And, of course, patients not admitted to intensive care still receive proper, compassionate, palliative care.

Opinions will differ as to which model is most helpful. On the one hand, too much flexibility may compromise the possibility of invoking a consistent process; and on the other, highly detailed scoring systems may disguise the reality of the need to make judgements in context which interweave ethical value and clinical experience. Whichever is preferred, it must be based on candour and trust between clinicians and their patients and this merits further discussion.

## Conclusions

The pandemic has given rise to much discomfort about the kinds of decisions that clinicians and front-line healthcare workers would be required to make in allocating limited healthcare resources. These decisions have forced us to confront rationing of interventions on a scale never before required. In addition to being deeply morally disturbing, they have given rise to specific legal objections. Decision-makers may have been traumatised by the nature of the clinical challenge and by the risk of being called to account in law.

We have sought to clarify for clinicians and front-line workers a framework which echoes in many respects the ethical frameworks developed for CCG resource allocation decisions more broadly, and which, subject to certain constraints and formalised processes, the law has accommodated. This framework takes a procedural approach to these decisions, recognising their intractable difficulty, the importance of context and the respect that is owed to individual patients and the community as a whole.

The calls for national guidance have been warranted. Such guidance would undoubtedly help to settle the collective nerves, even if it is not strictly necessary for the procedural framework we have articulated. But these calls have largely failed to capture the central issue: what the guidance should look like, and what consistency in decision-making demands. Our account above provides that content.

More broadly, outside the context of the pandemic, features of the current debate have revealed how, for too long, the traditions of bioethics and law have focused their attention on the rights and obligations of individual patients and clinicians.^37 38^ This, given the history of the respective fields, is understandable, but times such as these illustrate the myopia of this approach. In bioethics, as in medical law, there have been some notable recent movements towards thinking about legal, ethical and policy obligations at the community and social level.^39–41^ In the context of a pandemic, these kinds of public, community or wider social considerations are the more important. To be clear, to think in broader community terms is not to abandon the individual and their moral and legal specialness—far from it. It is simply to recognise that individual-specific values emerge from, and are embedded within, the context of the wider community in which all individuals are located.

## Footnotes

Bearing in mind the ‘hard look’ scrutiny to which the test is now subject.^42^

This broad banding of patients has been applied in local triage decision-making frameworks, including the ‘Pandemic Ethics Framework: Treatment decisions in the setting of the COVID-19 pandemic’ developed by the Oxford University Hospitals NHS Foundation Trust (D Wilkinson, personal communication, 2020), and also within an ethics framework for decision-making during the COVID-19 pandemic developed by the Royal College of Paediatrics and Child Health.^43^
